# Global Strategies for the Prevention and Control of Infectious Diseases and Non-Communicable Diseases

**DOI:** 10.2188/jea.JE20160010

**Published:** 2016-04-05

**Authors:** Hiroki Nakatani

**Affiliations:** 1 Graduate School of Medicine, Center for Global Health, Osaka University, Suita, Osaka, Japan; 2 Super Global Initiatives, Keio University, Mita, Tokyo, Japan

**Keywords:** global health, Sustainable Development Goals (SDGs), global strategy, non-communicable disease, infectious disease

## Abstract

This article on global health reviews the environment surrounding health strategies and plans, as well as lessons learned from the first 15 years of the 21st century, followed by a discussion on the quest for a new paradigm for disease control efforts and challenges and opportunities for Japan.

## INTRODUCTION

The paradigm of global health is rapidly changing. In particular, disease-control communities are nervous about the future of global efforts, which have drastically reduced the disease burden related to major infections and maternal and child health. In this paper, the author first reviews the environment surrounding health strategies and plans, as well as lessons learned from the first 15 years of global health in the 21st century. Then, the quest for a new paradigm of disease-control efforts and challenges and opportunities for Japan are discussed. The article is based on a keynote speech presented at the 2015 Global Health Workshop of the Association of Pacific Rim Universities (APRU), which was hosted by Osaka University, on October 30–31, 2015, in Osaka, Japan.

## ENVIRONMENT SURROUNDING HEALTH STRATEGIES AND PLANS

The year 2015 marks the last year of the Millennium Development Goals (MDGs). This year’s Nobel Prize in Physiology or Medicine could not be more relevant to the conclusion of the MDGs era. Professor Satoshi Omura was awarded the Prize for his discovery of ivermectin, a very innovative and powerful drug for onchocerciasis, or river blindness. Until recently, it was a common experience at riversides throughout Western Africa to see a child guiding an elderly person blinded by onchocerciasis. A statue depicting this scene has been erected in front of the World Health Organization (WHO) main building. The second good example celebrating the closure of the MDG is Professor Youyou Tu’s receiving of the Prize for her discovery of artemisinin, a very powerful anti-malaria drug. The incidence of malaria is now declining year by year.

**Figure 1.**
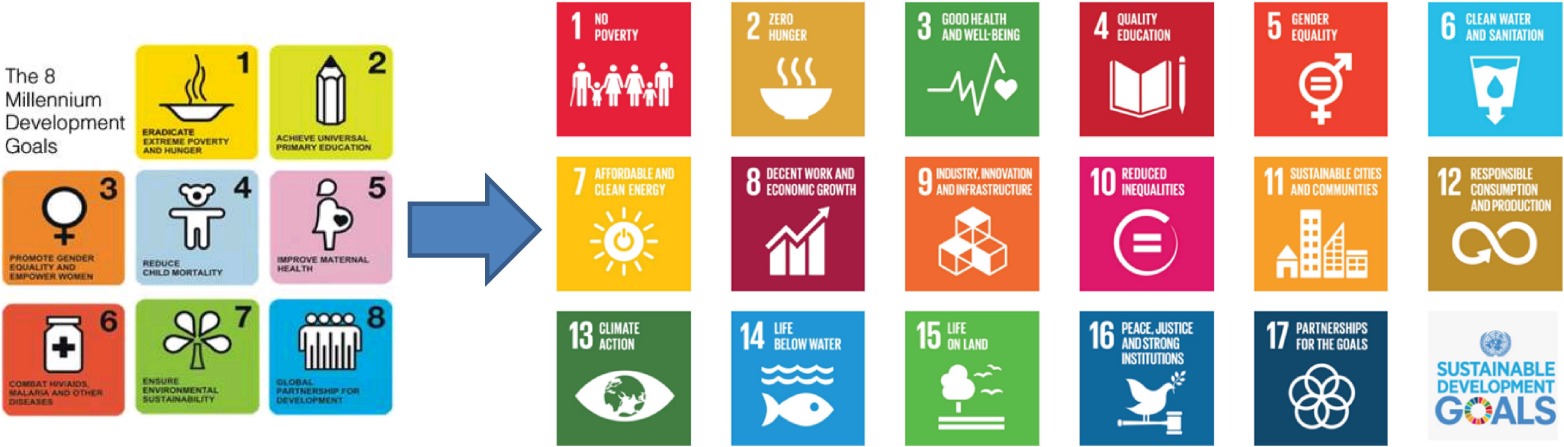
From MDGs to SDGs.

The transition from MDGs to Sustainable Development Goals (SDGs) is the central issue in the changing environment surrounding health strategies and plans. Figure 1 shows the change from MDGs to SDGs. In the health sector, the MDGs have been an internationally recognized framework for development and investment, and have proved their power. The Sustainable Development Summit at the United Nations (UN) headquarters adopted SDGs in September 2015. These became effective from January 1, 2016 and cover the period from 2016 to 2030. Health sectors are nervous about this transformation from MDGs to SDGs, and for good reasons.

The MDGs were relatively simple. Health occupied the central position, with health-specific MDGs accounting for three of a total of eight goals. One was MDG 4, to reduce child mortality, for which the targets were relatively simple: reduce the 5-year mortality rate by 2/3 between 1990 and 2015. MDG 5 aimed to improve maternal health. The target was to reduce the maternal mortality rate by 3/4 between 1990 and 2015. MDG 6 was to combat human immunodeficiency virus (HIV)/acquired immune deficiency syndrome (AIDS), malaria, and other diseases. The targets were to halt their increase by 2015, begin to reverse the spread of HIV/AIDS, and begin to decrease the incidence of malaria and other diseases. The goals were therefore clear and few, and the targets were relatively quantitative. This simple framework was agreed on and invested in by everybody.

In contrast, the SDGs have two characteristics. One is that they are more complex. Of 17 goals, only SDG 3, to ensure healthy lives and promote well being for all ages, specifically addresses health issues. As shown in Table 1, there are many targets under this single goal. The first three sub-targets are the continuation of MDGs 4, 5, and 6, in order to complete “unfinished business”. However, the other sub-targets seem to package previously unattended issues, such as substance abuse, traffic accidents, and universal health coverage. Many targets are not quantitative but qualitative. This proliferation of goals and targets arises from the participatory process used to develop the SDGs.^1^

**Table 1.** Goal 3: Ensure healthy lives and promote well-being for all at all ages

•	By 2030, reduce the global maternal mortality ratio to less than 70 per 100 000 live births
•	By 2030, end preventable deaths of newborns and children under 5 years of age, with all countries aiming to reduce neonatal mortality to at least as low as 12 per 1000 live births and under-5 mortality to at least as low as 25 per 1000 live births
•	By 2030, end the epidemics of AIDS, tuberculosis, malaria and neglected tropical diseases and combat hepatitis, water-borne diseases and other communicable diseases
•	By 2030, reduce by one third premature mortality from non-communicable diseases through prevention and treatment and promote mental health and well-being
•	Strengthen the prevention and treatment of substance abuse, including narcotic drug abuse and harmful use of alcohol
•	By 2020, halve the number of global deaths and injuries from road traffic accidents 3.7 By 2030, ensure universal access to sexual and reproductive health-care services, including for family planning, information and education, and the integration of reproductive health into national strategies and programmes
•	Achieve universal health coverage, including financial risk protection, access to quality essential health-care services and access to safe, effective, quality and affordable essential medicines and vaccines for all
•	By 2030, substantially reduce the number of deaths and illnesses from hazardous chemicals and air, water and soil pollution and contamination
•	Strengthen the implementation of the World Health Organization Framework Convention on Tobacco Control in all countries, as appropriate
•	Support the research and development of vaccines and medicines for the communicable and no communicable diseases that primarily affect developing countries, provide access to affordable essential medicines and vaccines, in accordance with the Doha Declaration on the TRIPS Agreement and Public Health, which affirms the right of developing countries to use to the full the provisions in the Agreement on Trade Related Aspects of Intellectual Property Rights regarding flexibilities to protect public health, and, in particular, provide access to medicines for all
•	Substantially increase health financing and the recruitment, development, training and retention of the health workforce in developing countries, especially in least developed countries and small island developing States
•	Strengthen the capacity of all countries, in particular developing countries, for early warning, risk reduction and management of national and global health risks

In addition, there is a basic change in concept between the MDGs and SDGs. SDGs are not only for developing countries and official development assistance (ODA) communities, but for all countries, both developed and developing. Therefore, SDGs are for everyone. The goals are categorized into five groups, the so-called 5Ps: People, Planet, Partnership, Peace, and Prosperity, which need to be promoted by all countries to create a sustainable world.

Another feature is that the SDGs emphasize linkage between goals more strongly than the MDGs. Health is not a monopoly of the health sector; interlinkage is very important. A typical example is onchocerciasis, an indigenous parasitic disease which occurs at riversides, particularly in West Africa. In the past, this disease often led to the abandonment of rich riverside farmland. But now, after regular mass treatment, people can return to these farming areas and resume farming. According to a World Bank estimate, the recovered farmland is producing food for a population of 17 million. Hence, control of onchocerciasis has contributed to the alleviation of hunger through food production and income generation. Health contributes to other goals and vice versa. Other examples are water quality and gender equality, both of which have a positive synergy with health. Accordingly, the SDGs represent a unique environment within which all health strategies and plans are required to work for the next 15 years.

## LESSONS LEARNED FROM THE FIRST 15 YEARS OF THE 21st CENTURY

In the first 15 years of the 21st century, global health has become a central agenda of international communities. After the 9/11 terrorist attacks, the G7 leaders felt it critical to address the root issues of terrorism by interrupting the vicious cycle of poverty and ill health. During this era, the number one disease burden for the working population in developing countries was HIV/AIDS, followed by tuberculosis. MDGs were already chosen, at the UN Assembly in 2000, and served as an excellent platform for attracting international commitments and resources to fight against poverty and ill health through investment in priority disease-control efforts. This led to the creation of the Global Fund to Fight AIDS, Tuberculosis and Malaria (Global Fund). Furthermore, two other important outcomes arose from the response to 9/11 which have shaped today’s global health landscape. One is strengthening of health security, triggered by the severe acute respiratory syndrome (SARS) experience in Asia in 2003. While 9/11 was physical terrorism, it is natural to be concerned about bio-terrorism as well. These combined concerns over terrorism and unusual new infections drove the world to drastically overhaul the quarantine system, resulting in the agreement of the International Health Regulations (IHR) public health treaty in 2005. Since then, all countries are requested to report unusual health events. Japan has reported a few incidents, such as the Tohoku Earthquake and Tsunami, the Fukushima nuclear accident in 2011, and more recently, dengue fever in 2014. WHO receives some 300 reports annually and assesses each for its significance to global health. Major influence also came from the report of the National Academy of America: Making the Nation Safer,^3^ which shaped the health research agenda for health security.

Reflecting these and related developments, health ODA has increased massively. Chris Murray^2^ studied the growth of health ODA and found that it increased from some 5 billion United States Dollars (USD) in 1990 to 27 billion USD in 2010. It should be noted that the composition of providers for this health ODA is changing. Resources from bilateral donors grew after 9/11, but contributions from international organizations remained quite modest. In contrast, the largest growth was observed from the private sector and from new funding mechanisms, such as the Global Fund, Global Alliance for Vaccines and Immunization, and the Bill and Melinda Gates Foundation (BMGF). The growing activities of non-state actors in global health are noteworthy. For example, the annual budgets of the BMGF and Médecins sans frontiers (MSF) are almost equal to the budget of WHO. Global health has evolved from the individual or philanthropic efforts of outstanding individuals and international organizations acting in this high-profile domain within international communities, and now represent a multi-polar field with many actors, particularly non-state actors.

These increased resources have been preferentially allocated to MDG 4, 5, and 6. The outcome appears in a special article of the Morbidity and Mortality Weekly Report,^4^ which listed the 10 greatest public health achievements worldwide from 2001 to 2010, as shown in Table 2. Specifically, AIDS-related mortality is down by 30% from its peak in 2005. Tuberculosis prevalence has fallen by 41%, and its mortality rate has fallen by 45% against the 1990 baseline. Malaria control is progressing. Four neglected tropical diseases—human African trypanosomiasis, lymphatic filariasis, blinding trachoma, and leprosy—are on track to be eliminated as public health problems, and guinea worm disease is also progressing towards eradication. The reduction in child mortality has also been remarkable: compared to approximately 12 million child deaths in 1990, only 6 million children died last year. Eighty percent of African children receive the basic vaccine package of BCG, polio, and DPT. Tobacco is one of the most serious epidemics, and its control is critical to prevent lung cancer and cardiovascular diseases. Tobacco control is making progress through an international convention called the WHO Framework Convention on Tobacco Control.

**Table 2.** Ten great public health achievements worldwide (2001–2010)

1.	Reductions in child mortality	6.	Tuberculosis control
2.	Vaccine-preventable diseases	7.	Control of neglected tropical diseases
3.	Access to safe water and sanitation	8.	Tobacco control
4.	Malaria prevention and control	9.	Increased awareness and response for improving global road safety
5.	Prevention and control of HIV/AIDS	10.	Improved preparedness and response to global health threats

## QUEST FOR A NEW PARADIGM

Table 3 lists the strategies and plans for communicable diseases and non-communicable diseases (NCD) adopted by the World Health Assembly. There are plenty of items related to communicable diseases, but relatively few for NCD. Why? Despite the fact that NCD represent the greatest challenge to global health, WHO has adopted few strategies and plans. Let us discuss the reason.

**Table 3.** Global strategies/plans adopted by the World Health Assembly (WHO’s governing body)

	Communicable diseases	Non communicable diseases
2015	WHA68.2 Global technical strategy and targets for malaria 2016–2030	

	WHA68.6 Global vaccine action plan	

	WHA68.7 Global action plan on antimicrobial resistance	

2014	WHA67.1 Global strategy and targets for tuberculosis prevention, care and control after 2015	

2013	WHA66.12 Neglected tropical diseases (including the Global Plan to Combat Neglected Tropical Diseases 2008–2015)	WHA66.8 Comprehensive mental health action plan 2013–2020

		WHA66.10 Follow-up to the Political Declaration of the High-level Meeting of the General Assembly on the Prevention and Control of Non-communicable Diseases (Including the global action plan for the prevention and control of noncommunicable diseases 2013–2020)

2012	WHA65.17 Global vaccine action plan	

2011	WHA64.14 Global health sector strategy on HIV/AIDS, 2011–2015	

2010		WHA63.13 Global strategy to reduce the harmful use of alcohol

In the communicable disease area, the Global Health Sector Strategy on HIV/AIDS 2011–2015 was adopted by the World Health Assembly in 2011. The adoption of this strategy carries much weight because it means that the health ministers of all 194 member countries agree with it. This is very important. This strategy defines who and how many to treat. Because the target set by the MDGs expired at the end of 2015, WHO started to produce new-generation strategies and plans aimed at the post-MDG era. A new strategy was adopted for tuberculosis in 2014 and for malaria in 2015. In 2016, a new strategy will be developed for HIV/AIDS. Strategy building is a resource-intensive process. WHO needs to consult with the member states, bring in scientific communities, and listen to civil societies. Informally, WHO has conversations with donors to determine whether they will financially support the proposed new strategy, in order that it be rendered a useful tool for affected countries, in terms of both resource mobilization and for the donors in terms of investment planning.

**Figure 2.**
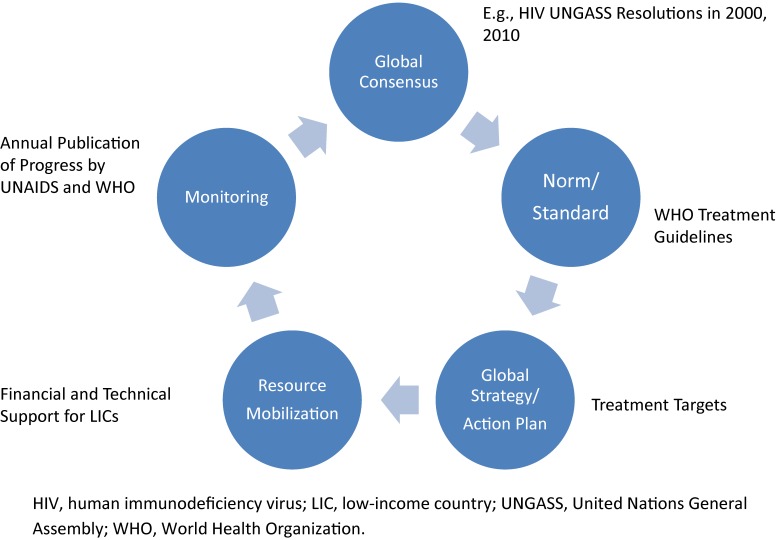
Global health cycle—the case of HIV.

Figure 2 shows my view of the global health circle, taking the example of HIV/AIDS, which has functioned very well to date. First, we need global consensus. In the case of HIV/AIDS, the UN General Assembly adopted relevant resolutions in 2000 and 2010. If you recall the year 2000, HIV treatment had become standard in the northern hemisphere, but patients in the southern hemisphere were left behind because the drugs were very expensive. The standard regimen used in most Western countries cost about 10 000 USD per year. This treatment gap attracted attention from political leaders and was discussed at the Kyushu-Okinawa Summit in 2000. However, political will alone could not solve the practical bottleneck of the high cost of the standard treatment regimen. Practical technical guidelines that accounted for the availability of technology and other resources in developing countries were lacking. At that time, HIV testing was expensive and difficult, and WHO faced the challenge of diagnosing HIV without sophisticated tests and treating patients with a cost-effective combination of generic drugs. WHO’s technical leadership was expected to overcome such challenges and see that the political commitments were successfully implemented in the field. This is a typical example of the norm/standard-setting function of WHO. Having established the norm and standard, we then need a global strategy to define how many patients to treat, how they should be treated, and when treatment should be initiated. Resources must then be mobilized to support the targeted disease-control efforts. Finally, monitoring is also very important, because sometimes resources are invested in the wrong place. For example, in many countries, the largest segment of health education on HIV still targets the general population, in which the likelihood of being affected by AIDS is relatively low. For different reasons, those who are at very high risk of HIV infection, such as sex workers, intravenous drug users, and the transgender population, are left behind. This is an important area where WHO and UNAIDS work in collaboration and blow whistles for public health authorities.

The control of neglected tropical disease (NTD) follows a slightly different cycle. The consensus is clear, with relevant resolutions adopted at the World Health Assembly. WHO has also developed various technical guidelines and road map,^5^ with clear targets to achieve by 2020. The unique part is resource mobilization. As the name NTD implies, the populations most affected tend to be marginalized and voiceless. Hence, little attention was given to this group of diseases until WHO and its partners bundled together 17 tropical diseases, which are not significant individually but collectively present serious public health and human rights challenges. The humanitarian gesture to help “the poorest among the poor” attracted the corporate social responsibility arms of major pharmaceutical companies, resulting in drug donation programs. Replacing direct ODA contributions, such drug donation programs are forming a unique private-public partnership in the case of NTD.

**Figure 3.**
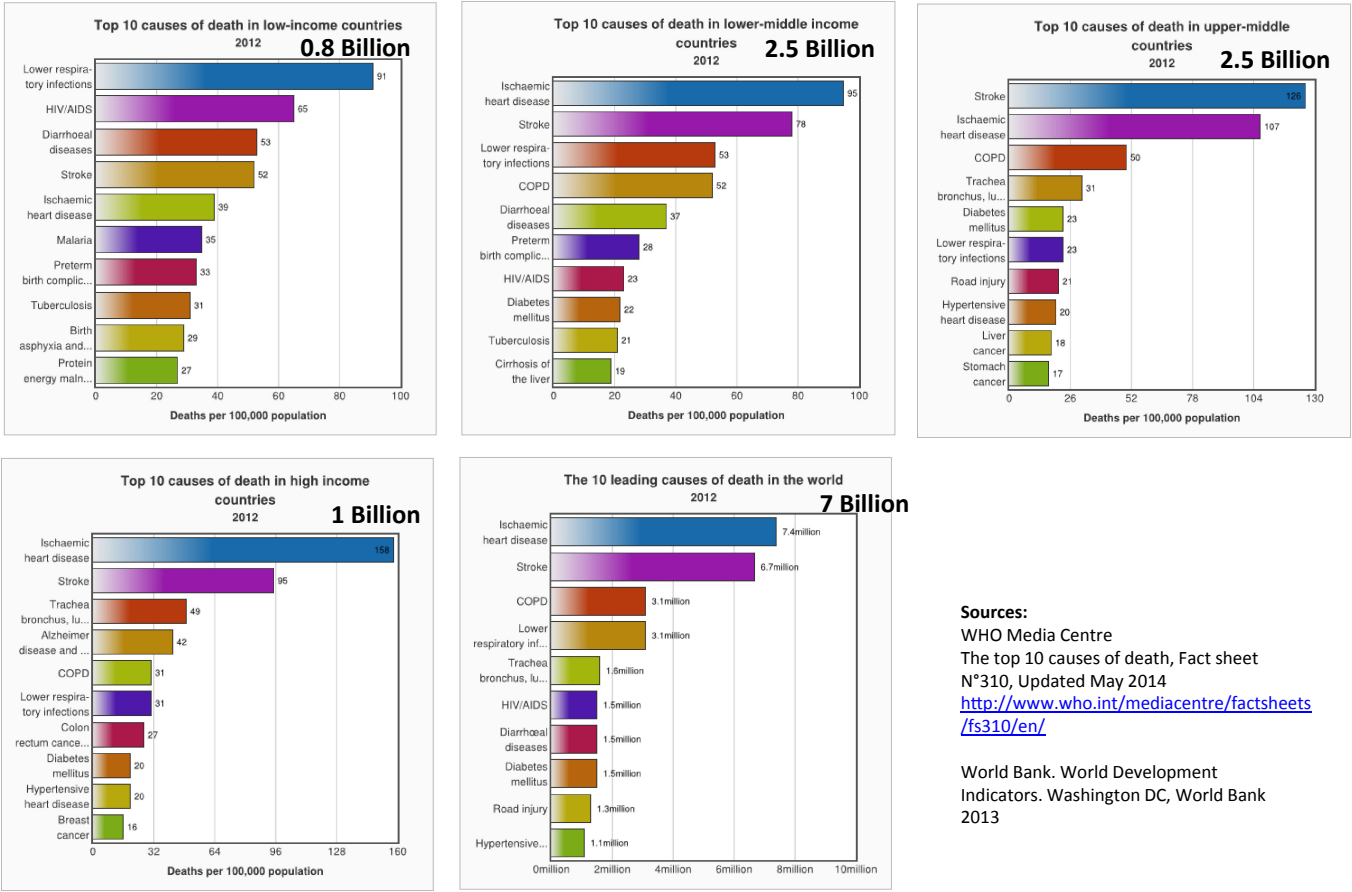
Top 10 causes of death by income category and population.

This global health cycle operated well for communicable diseases, such as HIV and NTD, which were designated goals in the MDGs era. What will be the new paradigm for emerging health challenges in the SDG era, covering 2016–2030? One needs to understand the health challenges that serve as parameters to shape our forthcoming global health. The first parameter is mortality. Figure 3 shows the top 10 causes of death in countries with different income levels. Worldwide, the three biggest killers are ischemic heart disease, stroke, and chronic obstructive pulmonary disease. However, 0.8 billion people live in low-income countries, where communicable diseases remain the major killers. On the other hand, the majority of the world’s population lives in middle-income countries, where morbidity patterns have shifted to non-communicable diseases and are closely similar to those in high-income countries.

The second parameter is population pattern. Life expectancy worldwide is 70 years, and has shown drastic improvements in Africa, Southeast Asia, and Latin America. The third parameter is prosperity. At the beginning of the 21st century, countries such as China and India were low-income countries, but have now become middle-income countries. China is the frontrunner, and India follows. Other resource-rich countries in the southern hemisphere may become high-income countries by 2020.

These changes are shaping the global health agenda and geopolitics. Reflecting the aging and affluence trends, causes of mortality are projected to include more cancer, ischemic heart disease, and cerebrovascular diseases, but less communicable diseases, including HIV and tuberculosis. Also, longevity necessitates long-term care, which is better assessed by disability-adjusted life years (DALY) instead of mortality. The DALY projections^6^ of WHO from 2004 for 2030 indicate that the number one disease burden will be depression, followed by ischemic heart disease.

Taking these changes in parameters in mind, the global health cycle for NCD control needs to be studied. In the case of hepatitis as a cause of hepatic cancer, linkage within the cycle is not complete, despite the clear consensus, expressed by the World Health Assembly Resolutions in 2010 and 2013, to expand hepatitis programs. The first challenge has been the development of guidelines for hepatitis C, because a new but highly expensive drug has come onto the market in high-income countries. For the Japanese market, one tablet costs about 500 USD, and patients need to take the tablets daily for 12 weeks. Whether to put this new drug in the guideline or to stick to the traditional interferon-based treatment regimen is controversial: everyone wishes to bring new drugs into the guideline, but if the treatment is not implementable, it will not be useful for practice. A second challenge is the development of a new global strategy for hepatitis, including treatment targets. Setting targets is difficult because the majority of heavily affected countries are middle-income countries, where ODA is not available. Hence, all resources should be mobilized domestically under the responsibility of national governments. Such responsibility will be scrutinized by monitoring organizations, which encourage governments to set targets low to avoid issues of government accountability. Linkage of technical components with financial realities is essential to any completion of this circle.

What is the status of the global health cycle for NCD? In 2012, NCD suddenly became a popular subject in the UN agenda. WHO was requested to make a global strategy and plan, bypassing guideline development, which requires a lot of work, such as simplifying the complicated treatment regimens of cancer. In addition, since NCD are most common in countries not eligible to receive ODA, as in the case of hepatitis, crafting strategies and setting treatment targets are very challenging. This is why the circle is not yet complete, and may account for the empty cells in Table 3.

While the SARS outbreak contributed to shaping global health policy in the last decade, the implications of the recent Ebola outbreak need to be explored. The statistics of the Ebola outbreak to date read nine countries infected, transmission across three continents, 26 000 cases, and 11 000 deaths, with the heaviest burden in three West African countries. Indeed, this is the largest Ebola epidemic but not the first. In previous outbreaks, patients were confined to remote villages, and the outbreak subsided after several dozen cases. However, this most recent outbreak was distinguished by the increased mobility within and between countries after the end of civil wars in some of the countries, and also the employment of many mobile workers by the booming mineral industry. These characteristics enhanced transmission into capital cities, where health infrastructure has not caught up with the growing population. WHO has been criticized for responding to the Ebola pandemic too late, handling it inefficiently, and coordinating poorly with other agencies. These shortcomings are partly the consequence of the shifting of priorities from communicable disease control to NCD control, which resulted in insufficient funding for the relevant programs. An independent review panel of WHO^7^ has identified the above issues and urged WHO to expedite reforms to better prepare for future epidemics. In addition, a high-level UN panel report and an Institute of Medicine report are coming soon. The major foci will be full implementation of IHR to detect signals of international health concerns, governance of response (including declaring global public health emergencies), as well as strengthening WHO’s global health infrastructure to support all these activities. Here, governance specifically means accountability, division of labor, and funding, and will require additional discussion at high policy levels. A test case for this is the division of labor in developing funding mechanisms currently hosted by WHO and the World Bank for funding reactions to large-scale epidemics. The former is called the WHO Contingency Emergency Fund^8^ and the latter is termed Pandemic Emergency Facilities.^9^

## CHALLENGES AND OPPORTUNITIES

This section will discuss the challenges and opportunities of these changes, as well as contributions from the Japanese public health community. For Japan, the greatest challenge is aging. Beginning in 2010, we have witnessed a gradual decline in the Japanese population, together with a sharp increase in the ratio of persons over 65 years old, particularly those exceeding 75 years. Japan has a very large population of centenarians aged over 100 years. Keio University has studied a cohort of centenarians^10^ and monitored their health status changes to investigate factors contributing to and impeding longevity. Indeed, longevity poses many challenges, not only for public health, but also for other social aspects. For example, persons with cognitive impairment face difficulties in making rational decisions, which are a fundamental aspect of our society. If we look at the prospect of population aging, more than 30% of the Japanese population already exceeds 60 years of age.

**Figure 4.**
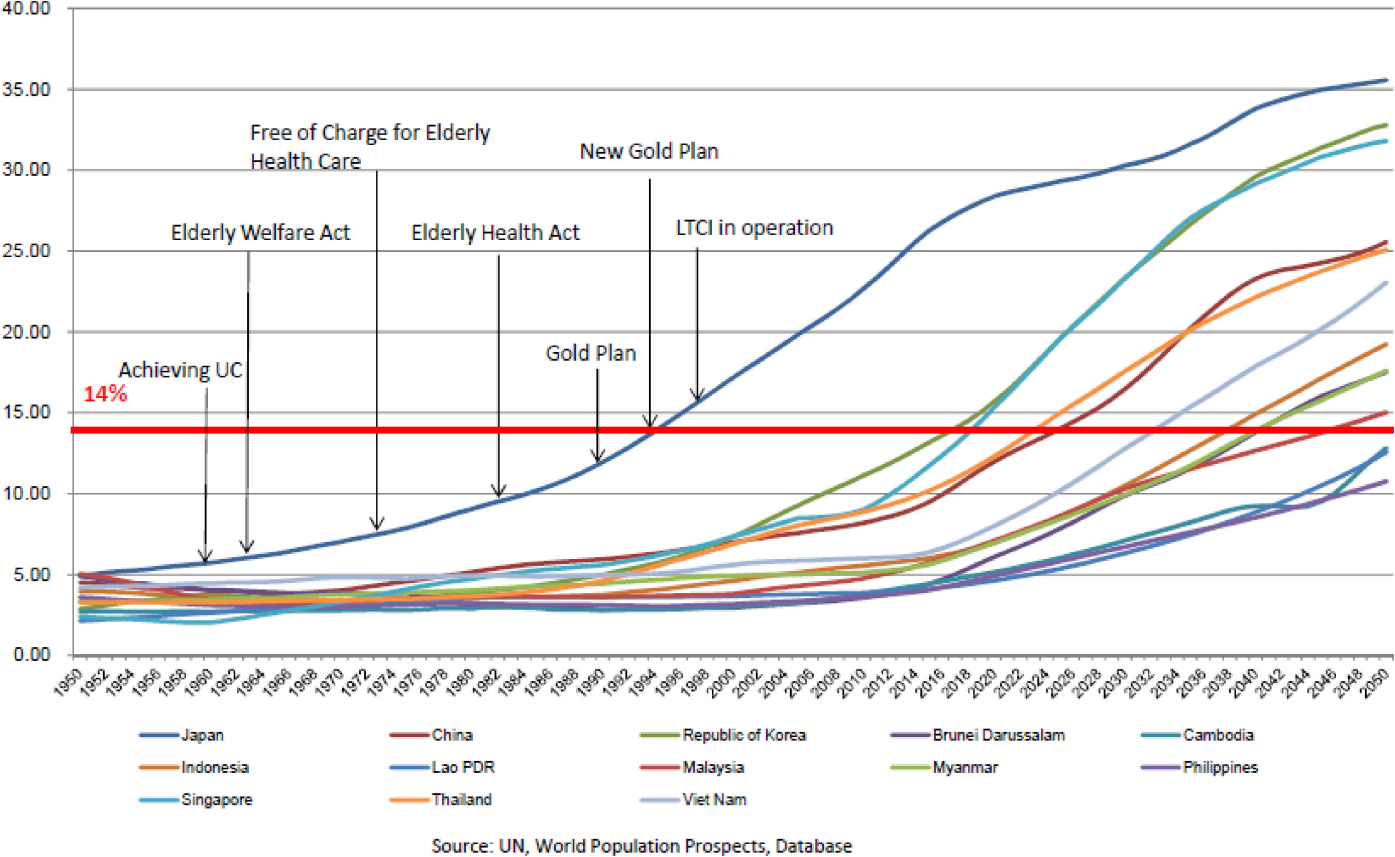
Aging rates of ASEAN countries and historical development of Japan’s elderly care system.^12^

Other Asian countries are following the same course, particularly the Republic of Korea, Singapore, and Taiwan. China and Thailand are aging rapidly as well. Such population aging is a global phenomenon, and not exclusive to Asia, Europe, and North America; Chile, for example, is also seeing remarkable aging of its population. In other words, aging is a common challenge for all APRU member countries. The Japanese experience of response to population aging can serve as a relevant case study for the world, Pacific Rim countries in particular. In the 1950s, the Japanese focus was on tuberculosis control. In 1952, 25% of the national medical expenditure was allocated to tuberculosis treatment, and 50% of all national hospital beds were occupied by TB patients. After a sharp decline in tuberculosis, Japan then transformed such service infrastructure to NCD control, particularly to the prevention and treatment of stroke. The Osaka University group,^11^ now headed by Professor Iso, has reported the evidence for public health interventions for stroke prevention and control. A second contribution from Japan could be our experience in policy development, as shown in Figure 4.^12^ Japan started to prepare for the growing elderly population from the 1970s. Our experience offers case studies of both success and failure. For example, free medical care for the elderly was introduced in 1973, triggered by a very populist governor. As governor of a big city with a small aging population, this politician thought that his policy was both affordable and sustainable. However, it soon became obvious that the free medical care program was very difficult to continue when the urban population aged and economic slowdown decreased tax revenue. Nevertheless, every politician who raised the possibility of discontinuing the program paid a great political cost at election time. The program was finally discontinued only after several elections.

Finally, innovations can turn challenges to opportunities. As discussed above, the world faces multiple challenges at the same time, and their solution requires novel approaches through innovations in public health. For example, the Medicine Patent Pool is expected to serve as a one-stop shop to handle complicated intellectual property and patent issues for the manufacture of generic drugs. Another important innovation is the various attempts to introduce different pricing policies for targeted populations based on their affordability. UNITAID is introducing innovative financing^13^ to reduce the prices of commodities by collecting an international solidarity levy.

How do all of these pieces come together to form a big picture? Japan has the unique opportunity in 2016 to host the G7 summit meeting and the Tokyo International Conference on African Development, which offers an international consensus-building forum for the new era of global public health. Already, Prime Minister Abe^14^ has published his firm commitment on global health through his contribution to The Lancet.

A new era of global public health is on the horizon.
